# Concentrations, Sources and Health Risk Assessment of Polycyclic Aromatic Hydrocarbons in Chinese Herbal Medicines

**DOI:** 10.3390/molecules29050972

**Published:** 2024-02-22

**Authors:** Deyan Cao, Zhu Zhu, Siyuan Zhao, Xi Zhang, Jianzai Lin, Junji Wang, Qinghong Zeng, Meilin Zhu

**Affiliations:** 1 School of Public Health, Ningxia Medical University, Yinchuan 750004, China; jolly2025@163.com (D.C.);; 2 Key Laboratory of Environmental Factors and Chronic Disease Control, Ningxia Medical University, Yinchuan 750004, China; 3 College of Basic Medical Sciences, Ningxia Medical University, Yinchuan 750004, China

**Keywords:** Chinese herbal medicine, polycyclic aromatic hydrocarbon, health risk assessment, Monte Carlo simulation

## Abstract

The determination and evaluation of 16 polycyclic aromatic hydrocarbons (PAHs) in seven Chinese herbal medicines (CHMs) were conducted through a rapid and straightforward extraction and purification method, coupled with GC-MS. A sample-based solid-phase extraction (SPE) pretreatment technique, incorporating isotopic internal standards, was employed for detecting various medicinal parts of CHMs. The assay exhibited linearity within the range of 5 to 500 ng/mL, with linear coefficients (R^2^) for PAHs exceeding 0.999. The recoveries of spiked standards ranged from 63.37% to 133.12%, with relative standard deviations (RSDs) ranging from 0.75% to 14.54%. The total PAH content varied from 176.906 to 1414.087 μg/kg. Among the 16 PAHs, phenanthrene (Phe) was consistently detected at the highest levels (47.045–168.640 μg/kg). Characteristic ratio analysis indicated that oil, coal, and biomass combustion were the primary sources of PAHs in CHMs. The health risk associated with CHMs was assessed using the lifetime carcinogenic risk approach, revealing potential health risks from the consumption of honeysuckle, while the health risks of consuming *Lycium chinense* berries were deemed negligible. For the other five CHMs (glycyrrhizae, *Coix lacryma*, ginseng, lotus seed, seed of *Sterculia lychnophora*), the health risk from consumption fell within acceptable ranges. Furthermore, sensitivity analyses utilizing Monte Carlo exposure assessment methods identified PAH levels in CHMs as health risk sensitizers. It is crucial to recognize that the consumption of herbal medicines is not a continuous process but entails potential health risks. Hence, the monitoring and risk assessment of PAH residues in CHMs demand careful attention.

## 1. Introduction

In contrast to synthetic drugs, which effectively treat various diseases but often come with side effects, Chinese herbal medicines (CHMs) have gained increasing interest in recent years due to their fewer side effects [1,2]. They have become widely accepted worldwide and are utilized by numerous pharmaceutical companies as a top-tier resource for discovering natural bioactive compounds [3]. According to estimates from the World Health Organization (WHO), over 80% of the global population primarily relies on traditional medicine, a substantial portion of which involves the use of plant extracts or their active ingredients [4]. The commercialization of medicinal plants has experienced significant growth, with herbs finding applications in various fields such as phytochemicals, pharmaceuticals, nutraceuticals, herbal remedies, food supplements, perfumes, cosmetics, and food flavoring [5]. However, a key issue in the development and application of herbal medicines is ensuring their quality and safety. This concern extends not only to toxic ingredients but also to residual contaminants, including pesticides [6,7,8], heavy metals [9,10], polycyclic aromatic hydrocarbons (PAHs) [11,12], and other environmental pollutants.

PAHs constitute a sizable group of semi-volatile organic compounds, characterized by two or more fused aromatic carbocycles, and are designated as environmental pollutants due to their carcinogenic properties [13,14]. Notably, the U.S. Environmental Protection Agency has identified 16 PAHs as priority control pollutants, commonly known as the 16 EPA-PAHs. The primary sources of PAHs stem from incomplete combustion and thermal decomposition of organic matter, encompassing fossil fuels and biomass [15,16,17,18]. This occurs predominantly when proteins, lipids, and carbohydrates undergo incomplete combustion at elevated temperatures (usually between 300 and 600 °C) [19]. Exposure to PAHs in the population, especially among non-smokers, occurs through various routes, including the atmosphere, water, air, food, and the food chain [20,21,22]. Studies have indicated that plants can accumulate PAHs from the environment, leading to the contamination of crops and herbs [23]. There are two primary pathways for PAH accumulation in plants [24]: one involves the uptake of PAHs from the soil through the plant’s root system, while the other involves the absorption of atmospheric PAHs through the above-ground parts of the plant [25]. Consequently, these processes result in the accumulation and presence of harmful substances in Chinese medicinal plants, significantly impacting the quality of CHMs. PAHs have been associated with a range of adverse health effects, potentially disrupting male and female hormones, causing endocrine disruption, infertility, and immunosuppression, as well as exhibiting carcinogenic and teratogenic properties [26,27,28]. It is crucial to safeguard herbal medicines and herbal products from such contaminants, ensuring they are either protected or controlled for use within safe levels [29]. Given the increasingly stringent testing standards set by the European Union, Japan, and other countries, international organizations have yet to formulate a fully unified standard for CHMs. Therefore, there is a pressing need to establish a method based on the detection and analysis of harmful substances like PAHs in CHMs, coupled with health risk assessments.

## 2. Results and Discussion

### 2.1. Optimization of Extraction Conditions

**Figure 1:**
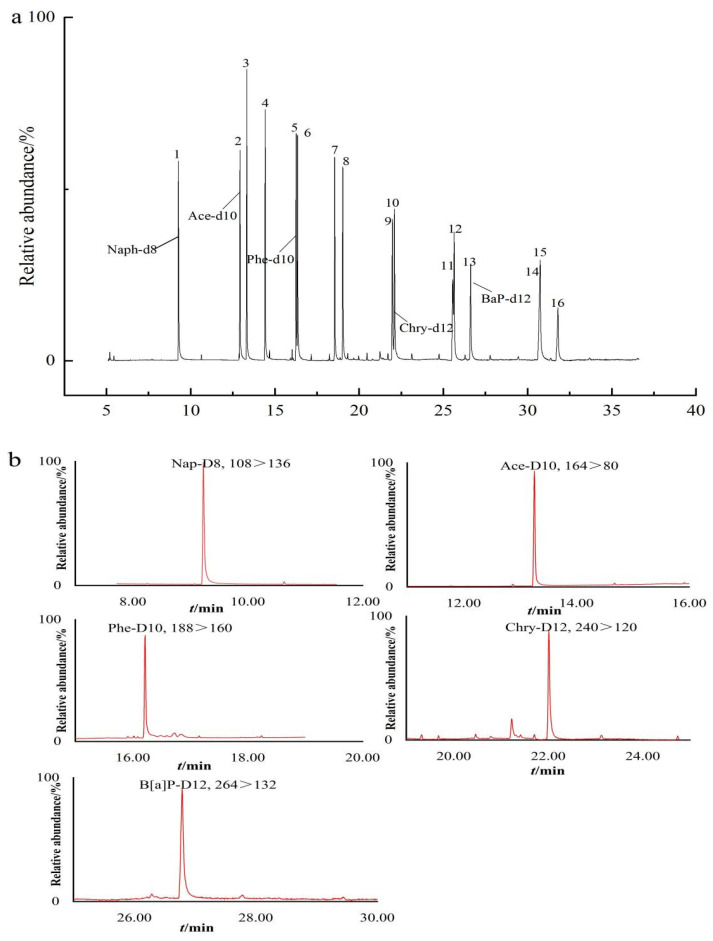
TIC of PAH standard (**a**) and their deuterated PAH standard (**b**). Naphthalene (1), Acenaphthylene (2), Acenaphthene (3), Fluorene (4), Phenanthrene (5), Anthracene (6), Fluoranthene (7), Pyrene (8), Benz[a]anthracene (9), Chrysene (10), Benzo[b]fluorathene (11), Benzo[k]fluorathene (12), Benzo[a]pyrene (13), Indeno[1,2,3-c,d]pyrene (14), Dibenz[a,h]anthracene (15), Benzo[g,h,i]perylene (16). Peak identification numbers correspond to compounds reported in Table 1.

GC-MS offers high chromatographic resolution, with mass spectra providing elevated mass selectivity and a wealth of structural information. In this experiment, an Agilent DB-5 capillary column was employed to explore various temperature ramping programs. Based on the test results, we opted to use the earlier-described temperature program, allowing for baseline separation and sample detection within a 32 min timeframe. Furthermore, GC-MS, utilizing an electron ionization energy of 70 eV, was our instrument for the quantitative analysis of PAHs. Figure 1 illustrates the total ion chromatograms of the blank matrix with added target analytes and each internal standard. The data in Figure 1 and Table 1 indicate consistent retention times for standards and their isotopes, with no matrix interference. Isomers (Phe/Ant, Flt/Pyr, BaA/Chry, and BbF/BkF/BaP) could be baseline separated within the 32 min window.

The experiment investigated ultrasonic extraction and oscillatory extraction as pretreatment methods, utilizing acetonitrile and n-hexane/acetone as extraction solvents for comparison. The effectiveness of the three parallel methods was assessed through recovery comparisons. Our outcomes demonstrated that ultrasonic and acetonitrile extraction proved more effective compared to oscillatory and n-hexane/acetone extractions. While ultrasonic extraction outperformed oscillatory extraction, recoveries for analytes with small molecular weights (Naph, Acy, Ace, Flu) were not particularly high, necessitating repeated extraction or a change in the extraction solvent. Analytes with low recoveries, likely due to their volatility and low boiling points, were possibly lost during evaporation. None of the analytes exhibited a relative standard deviation (RSD) exceeding 15%. Table 2 presents the recoveries of the 16 PAHs (with 5 PAHs-D as a surrogate, *n* = 6) in the seven CHMs, showcasing recovery ranges for the three spiked concentrations (*n* = 6) from 63.37% to 133.12%.

### 2.2. Performance of the Method

To ensure experimental accuracy, we utilized an analytical calibration curve. Regression equations were derived using five-point concentrations ranging from 5 to 500 ng/mL. As shown in Table 3, the 16 PAHs exhibited favorable linearity, with a correlation coefficient (R2) of 0.999. The limit of detection (LOD) and limit of quantification (LOQ) were determined by adding different amounts of mixed standards to blank samples, and the signal-to-noise ratio (S/N) ≥ 3 was used to determine the LOD, and the signal-to-noise ratio (S/N) ≥ 10 was used to determine the LOQ. The LODs for the 16 analytes ranged from 4.96 to 8.14 μg/kg, and the LOQs ranged from 9.9 to 16.3 μg/kg (Table 3).

### 2.3. Application to Real Samples

**Figure 2:**
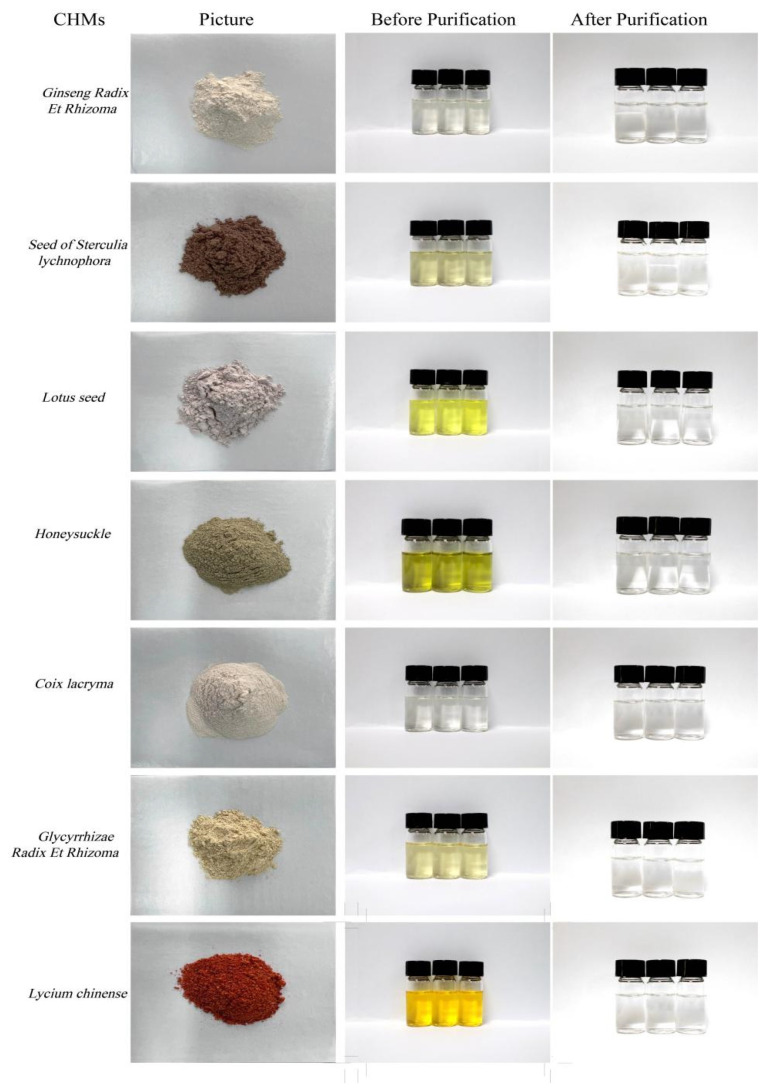
CHMs of seven types and the purification performances compared before and after cleanup.

The aforementioned method was applied to monitor the presence of 16 PAHs in seven CHMs, yielding results with an RSD of less than 15%. Calibration curves for mixed working standard solutions were constructed by plotting the peak area ratios of the quantitative ion pairs of each standard substance to the five internal standard substances. Extracts were analyzed by GC-MS, and the outcomes are detailed in Table 4. The composition of herbs is extremely complex, with a wide range of amino acids, volatile oils, sugars, and vitamins. Currently, Florisil solid-phase extraction (SPE) columns, PSA SPE columns, and C18 SPE columns are used for cleanup in many studies. PSA can chelate with metal ions and can effectively remove fatty acids, organic acids, and some polar colors and sugar substances. The functional group of C18 contains 10% carbon, has hydrophobic effect, and has adsorption effect on neutral and non-polar components, such as aromatic oils and fat-soluble vitamins. PSA filler removes water, coloring, and other substances. The experimental purification was carried out using a combination of three commonly used adsorbents to investigate the effect of purification of herbal medicines, the purification effects of the seven herbs are depicted in Figure 2.

The contamination levels of the 16 EPA-PAHs were assessed in seven selected CHMs at varying levels, revealing contamination rates of 62.50% for honeysuckle, 56.25% for the *seed of Sterculia lychnophora*, 68.75% for glycyrrhizae, 35.83% for *Coix lacryma*, 37.50% for ginseng, 50% for lotus seed, and 37.50% for *Lycium chinense* berries. Statistically different contamination rates of PAHs in seven Chinese herbal medicines (chi-squared test χ^2^ *p* < 0.05). Glycyrrhizae exhibited the highest contamination, while *Coix lacryma* showed the least contamination. The contamination rates were ranked from high to low, as follows: glycyrrhizae > honeysuckle > seed of *Sterculia lychnophora* > lotus seed > ginseng > *Lycium chinense* berries > *Coix lacryma*. Upon analyzing the total detected concentrations of each herbal medicine type, root and stem samples had the highest levels, followed by flowers, while fruit samples exhibited the lowest levels, and similar trends were observed in the TEQ. This may be due to the fact that root herbs are exposed to soil for a long period of time relative to flowers and fruits and absorb PAHs through soil and water, resulting in a large amount of PAHs being enriched in the roots, where the highest mass concentrations of PAHs were found. Fruits and seeds, on the other hand, develop later, are exposed to PAHs in the environment for a shorter period of time, and are enriched with low amounts of PAHs. In addition, different types of herbs may come from different places of origin, so the concentration of PAHs in the environment of the herbs’ place of origin is another important factor affecting the concentration of PAHs.

Furthermore, concentration levels of individual PAHs in different herbs were analyzed. Among the 16 PAHs, Phe emerged as the most prevalent and severe contaminant, displaying the highest contamination levels and was detected in all tested samples, with total mass concentrations of PAHs-Phe ranging from 47.045 to 797.061 µg/kg. Notably, PHAs-Ant and Naph also exhibited higher concentration ranges, with values of nd~653.492 µg/kg and 21.666~287.545 µg/kg, respectively. BbF was detected in only three herbs (glycyrrhizae, honeysuckle, and the seed of *Sterculia lychnophora*), with the highest level recorded at 941.504 µg/kg. BaP, a representative carcinogenic PAH, was found only in *honeysuckle* at 39.928 µg/kg. From the results of PAH assay monomers, it was found that lower molecules of PAHs had higher levels of contamination in herbal medicines, which was related to the higher solubility and faster transfer rate of low-ring PAHs from soil to plants.

In 2017, China’s National Standard for Food Safety set limits for BaP in various food products, with a 5 µg/kg limit for cereals and their products, meat and meat products, aquatic animals and their products, and oils and fats and their products. *Honeysuckle* exceeded this limit, and European Union regulations also established limits for PAHs (PAH4) at 35 µg/kg. Glycyrrhizae, honeysuckle, and the seed of *Sterculia lychnophora* were also found to exceed these limits, as indicated by (EU) no. 1, Standard No. 835/2011 for different food products, such as processed cereals or dietary foods for special medical purposes intended for infants.

### 2.4. Distributional Characteristics of PAHs

**Figure 3:**
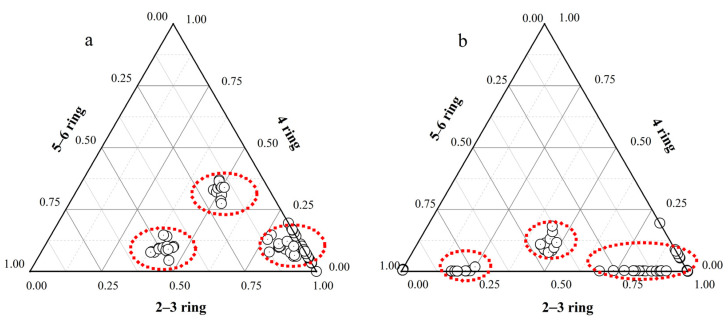
Distribution of PAHs with different ring numbers in herbal medicine samples. (**a**) Concentration percentage. (**b**) Toxic equivalent concentration percentage.

Figure 3 illustrates the distribution of PAHs with different ring numbers based on concentration and TEQ, respectively. A comparison between the two reveals a discrepancy (*p* < 0.05). When considering total concentration for analysis, PAHs with 2–3 rings predominated, representing over 60% of 4-ring PAHs in most samples. PAHs with larger molecules (5–6 rings) accounted for less than 10% of all samples, aligning with findings reported by Ishizaki and Kataoka [1]. In other words, small-molecule PAHs were predominant among the detected PAHs in the CHMs. In contrast, when evaluated using toxic equivalent concentration (Figure 3b), no large-molecule PAHs were detected. The percentage of 5–6-ring PAHs in the samples was 0, and the percentage of total toxic equivalents of 2–3-ring PAHs exceeded 90%. This outcome can be attributed to the higher toxicity of large-molecule PAHs compared to small-molecule PAHs. However, the detection rate of large-molecule PAHs was low, resulting in a higher overall toxic equivalent concentration for small-molecule PAHs. The substantial proportion of low-molecular-weight (2–3-ring) PAHs suggests the potential presence of recent contamination in the affected area. Herbs not only actively absorb harmful pollutants in the soil during the growth process, but may also passively absorbing pollution caused by anthropogenic activities such as haze weather that emits PAHs.

### 2.5. PAH Source Analysis

Natural sources of PAHs encompass volcanic eruptions and brush burning, which directly contributes to background PAH levels in the environment. However, the primary origins of PAHs are anthropogenic, stemming from incomplete combustion of petroleum fuels, biomass, and the natural volatilization or leakage of petroleum fuels [30]. The diverse range of PAH sources renders them ubiquitous environmental pollutants. Analyzing the sources of PAHs in CHMs is crucial, not only for understanding the pollution origins, but also for effective PAH control. Several methods, such as the characteristic marker method, characteristic compound ratio method, and multivariate statistical method, are employed for source analysis. Among these, the characteristic compound ratio method is frequently utilized.

This method relies on significantly different concentration ratios of PAHs from various pollution sources. Due to the volatility of PAHs in the gas phase, the ratio method primarily utilizes data from PAHs adsorbed on particulate matter to infer potential sources. Four ratios distinguish PAH sources from crude oil pollution, gasoline combustion, biomass combustion, and coal combustion. The judgment criteria involve the dominance of 2–3-ring PAHs, indicating petroleum source pollution, while the dominance of four or more rings PAHs suggests high-temperature combustion sources. In this study, the herbal samples were judged to be primarily influenced by petroleum sources of PAHs [31,32,33,34,35,36]. The monomer distribution of PAHs provides insights into their potential sources. Three characteristic ratios—Flt/(Flt + Pyr), BaA/(BaA + Chr), and Ant/(Ant + Phe)—were employed in this study to characterize the pollution sources of CHMs.

**Figure 4:**
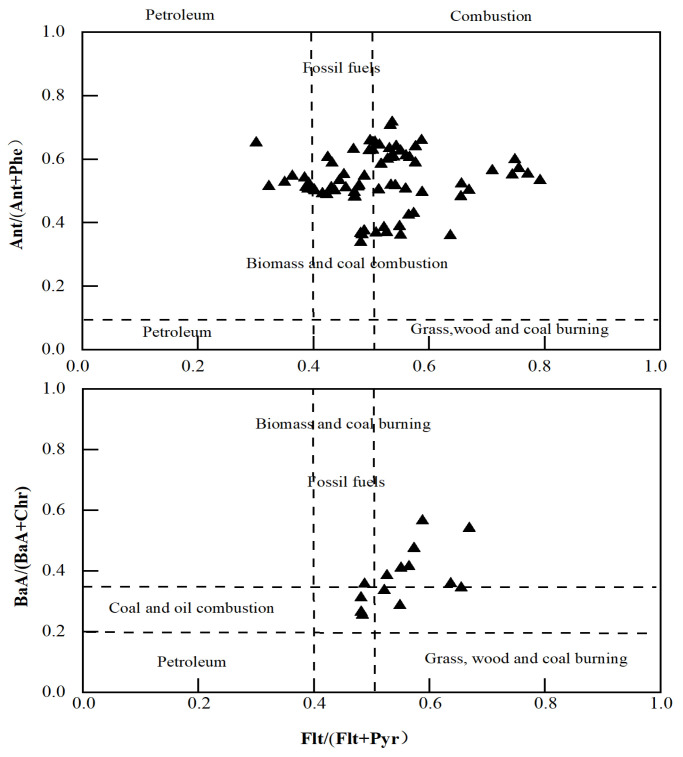
Crossplot of Flt/(Flt + Pyr), BaA/(BaA + Chr) and Ant/(Ant + Phe) characteristic ratios.

Empirical results for seven CHM samples indicated that Flt/(Flt + Pyr) ratios mainly ranged between 0.4 and 0.60, reflecting characteristics of biomass and fossil fuel combustion, as well as vegetation, grass, and coal combustion sources. BaA/(BaA + Chr) ratios exceeding 0.2 exhibited dual characteristics of oil-fired and biomass-fired coal, while Ant/(Ant + Phe) ratios greater than 0.1 displayed dual characteristics of biomass-fired coal and fossil fuels. Overall, these characteristic ratios suggested that the main sources of PAHs in CHMs are combustion sources, including biomass combustion and coal combustion. Plotting crossplots BaA/(BaA + Chr), Flt/(Flt + Pyr), and Ant/(Ant + Phe) (Figure 4) helped us to visualize the possible sources of PAH contamination in traditional Chinese medicine. Data points from the characteristic ratios indicated that oil sources, biomass, and coal combustion collectively contribute to herbal medicine pollution, posing significant environmental impacts and constituting the most substantial pollution source among the identified sources. However, the vast variety of herbal medicine types, coupled with the absorption, enrichment, transportation, and transformation of PAH compounds within the plant [37], as well as potential production and processing-related PAH production, necessitate further investigation to determine the precise sources of PAHs in CHMs.

### 2.6. Health Risk Assessment

**Figure 5:**
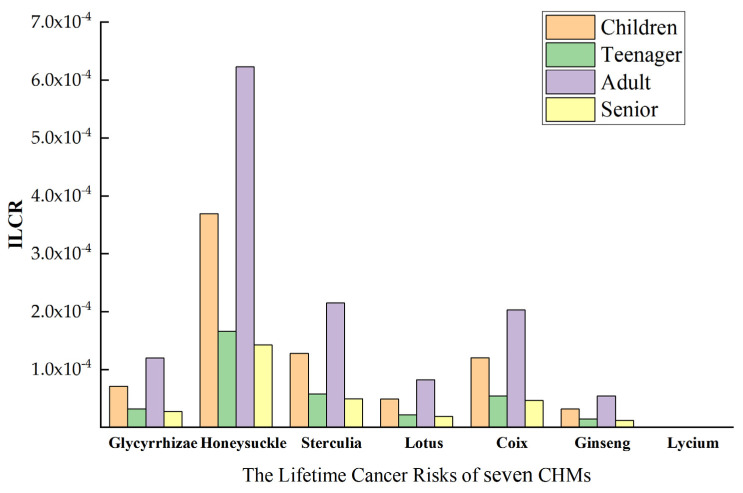
Health risk assessment of herbal medicines (ILCR).

PAHs are commonly detected in CHM samples, and their potential human health risks should not be ignored. The results of the ILCR evaluation of PAH contamination in seven CHMs are presented in Figure 5 and Table S3. Except for *Lycium barbarum*, the ILCR health risk values associated with the intake of herbs across different age groups primarily fell within the range of 1.0 × 10^−6^ to 1.0 × 10^−4^, indicating a potential carcinogenic risk. However, the calculated risk levels remained within the acceptable range. Notably, in four age groups, the carcinogenic risk linked to the ingestion of *Lycium barbarum* was negligible (ILCR < 1.0 × 10^−6^). Although the health risk of PAHs to humans is within acceptable limits, the potential carcinogenic risk should be emphasized and the quality and safety control of traditional Chinese medicines need to be further strengthened.

Regarding the extent of PAH contamination in CHMs, statistically significant differences in cancer risk among seven Chinese herbs (*p* < 0.05), and the order of carcinogenicity risk is as follows: honeysuckle > *Coix lacryma* > lotus seed > glycyrrhizae > ginseng > seed of *Sterculia lychnophora* > *Lycium chinense*. This suggests a higher risk of carcinogenicity associated with the consumption of honeysuckle as a raw material for CHMs and its products. As a consequence, there is a need to enhance the detection and control of residual PAH contamination in honeysuckle raw materials. The highest cancer risk from honeysuckle consumption in adults may be attributed to prolonged dietary exposure to herbs among the analyzed parameters. Moreover, research indicates that children exhibit greater sensitivity to pollutants, posing health risks, underscoring the importance of prioritizing children’s health concerns in this context [38].

Table 5 shows the results of the probabilistic assessment of the Monte Carlo carcinogenic risk of herbal medicines. To determine the carcinogenic risk, a lognormal distribution was fitted to the ILCR values of the herbs. The 90th percentile ILCR values for glycyrrhizae, *Coix lacryma*, ginseng, lotus seed, seed of *Sterculia lychnophora*, and *Lycium chinense* were 4.69 × 10^−5^, 1.48 × 10^−4^, 1.20 × 10^−4^, 1.45 × 10^−4^, 5.92 × 10^−4^, and 2.25 × 10^−6^, respectively. None of these values exceeded the maximum acceptable level of 1.0 × 10^−4^. However, the 90th percentile ILCR value for *honeysuckle* was 1.37 × 10^−3^, surpassing the maximum acceptable level. Consequently, it can be inferred that the intake of the herbal medicines analyzed in this study by residents leads to varying degrees of carcinogenic risk, with significant differences in risk levels (*p* < 0.05); however, no unacceptable carcinogenic risk occurred.

**Figure 6:**
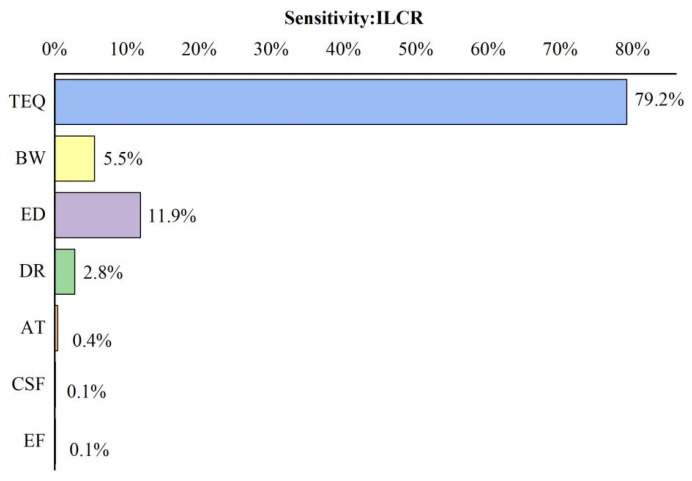
Sensitivity analysis of the carcinogenic risk of PAHs ingested from CHMs.

The sensitivity analysis results, presented in Figure 6, highlight the predominant factors influencing total carcinogenic risks associated with herbal medicines. The TEQ of PAHs emerged as the most influential factor, contributing significantly to health risks at 79.2%. Following closely was the exposure duration (ED) related to the dietary exposure time to herbal medicines, accounting for 11.9% of the total risk. The findings from the sensitivity analysis underscore TEQ as the most critical factor, emphasizing that the residual amount of PAHs in Chinese herbal medicines plays a pivotal role in shaping the overall carcinogenic risk. High enrichment of PAHs in herbal medicines increases the likelihood of human intake and increases the risk of human dietary exposure; therefore, the concentration of PAHs in herbal medicines is a key factor in the development of carcinogenic risk. This suggests that effective monitoring of PAH concentrations in herbal medicines could prove instrumental in mitigating the health risks posed by PAHs to the population.

## 3. Experimental Methods

### 3.1. Instrumentation

PAHs were separated on an Agilent DB-5MS capillary column (60 m × 0.25 mm × 0.25 μm), using a 7890-5977 Gas Chromatography Mass Spectrometer (GC-MS) (Agilent, Santa Clara, CA, USA). The flow rate of the column was 1.5 mL/min; the temperature of the injection port was 300 °C; the temperature of the interface was 310 °C; the ion source was 250 °C; the four-stage rod was 150 °C; the injection volume was 2 μL; the split ratio was 5:1; and the solvent excision time was 5 min (determined according to the peak time of cyclohexane). The measurement was performed in SIM mode. Temperature increase procedure: 80 °C for 0.5 min, then 10 °C/min to 200 °C for 0 min, then 20 °C/min to 260 °C for 0 min, then 5 °C/min to 290 °C for 2 min, then 5 °C/min to 315 °C for 8 min. Other instruments used for sample preparation are as follows: 8000C Multi-function pulverizer (Yongkang Red Sun Electromechanical Co., Ltd., Zhejiang, China), AL204 Electronic balance (METTLER TOLEDO INSTRUMENTS, Shanghai, China), Sorvall ST 16R High-speed Freezing Centrifuge (Thermo Fisher, Waltham, MA, USA), VJL-Eortex Mixer (Shanghai Jinlan Instrument Manufacturing Co., Shanghai, China), HGC-96ANitrogen Blower (Tianjin Hengao Technology Development Co., Tianjin, China), and KQ5200DE Type ultrasonic instrument (Kunshan Ultrasonic Instrument Co., Kunshan, China). The optimized parameters for the analysis of 16 PAHs and 5 deuterated PAHs (PAHs-D) using GC-MS with selected ion monitoring mode (SIM) are listed in Table 1.

### 3.2. Chemicals and Solutions

The 16 PAHs’ standard mixture (1000 mg/L, purity ≥ 96%, hexane/acetone 1:1) and 5 PAHs’ isotope internal standard mixture (2000 mg/L, purity ≥ 98% methylene dichloride) were purchased from ANPEL Laboratory Technologies Inc. (Shanghai, China), including naphthalene, acenaphthylene, acenaphthene, fluorene, phenanthrene, anthracene, fluoranthene, pyrene, chrysene, benzo[a]anthracene, benzo[b]fluoranthene, benzo[k]fluoranthene, benzo[a]pyrene, indeno[1,2,3-cd]pyrene, dibenzo[a,h], anthracene, benzo[g,h,i]perylene, naphthalene-d8, acenaphthene-d10, phenanthrene-d10, chrysene-d12, and perylene-d12. PAH standard and isotope internal standard stock solutions were diluted in cyclohexane at a concentration of 10 µg/L. Three types of SPE columns, ProElut C18 (1 g/6 mL), ProElut Florisil (1 g/6 mL), and ProElut PSA (500 mg/6 mL) (Dikma Technologies, Radnor, PA, USA), were used for sample purification. All organic solvents, including acetonitrile, cyclohexane, and ethyl acetate were of HPLC grade (Dikma Technologies Inc., Radnor, PA, USA).

### 3.3. Sample Pretreatment

Thirty-five samples of CHMs (ginseng radix et rhizoma, seed of *Sterculia lychnophora*, lotus seed, honeysuckle, *Coix lacryma*, glycyrrhizae radix et rhizoma, *Lycium chinense*; 5 each) were purchased and analyzed, all of which were locally grown and harvested. The samples were stored in a cool, ventilated, and dry environment after purchase from the local market. Individual samples were weighed appropriately (>200 g) and crushed into powder using a high-speed pulverizer, which passed through a 60-mesh sieve, then collected into labeled sample bags and stored at −20 °C for further analysis.

#### 3.3.1. Extraction

Weigh 0.500 g of herbal powder in a 15 mL centrifuge tube; add 100 μL of mixed isotope internal standard; mix thoroughly; add 5 mL of acetonitrile; seal with a lid; carry out ultrasonic extraction for 30 min; leave it to cool and then centrifuge it at 4000 r/min for 5 min. Transfer the supernatant to a clean 15 mL centrifuge tube. Precipitate, then add 5 mL of acetonitrile. Carry out ultrasonic extraction once again, followed by centrifugation, then combine the supernatant. Add 3 g of anhydrous sodium sulfate to absorb water by sufficient shaking. Perform centrifugation once again; take the extracted liquid nitrogen, blowing at 40 °C to a volume of about 0.2 mL, then add 2 mL of cyclohexane to dilute and dissolve, and then proceed to the next step of purification. Extraction of PAHs and the following purification methods reference the methods of Cui, Z. et al. [39].

#### 3.3.2. Purify

For root and stem samples (ginseng radix et rhizoma, glycyrrhizae radix et rhizoma) the extraction solvent was cyclohexane, the purification column was PSA, and the activation and elution solution was cyclohexane. For the fruits and seeds (seed of *Sterculia lychnophora*, lotus seed, *Coix lacryma*, *Lycium chinense*) the purification column was a C18 column, activated with cyclohexane, with acetonitrile as eluent. For flowers (*honeysuckle*), the purification columns were C18, Florisil, PSA, acetonitrile, cyclohexane–ethyl acetate (49:1, *v*/*v*), and cyclohexane as eluents. Purification process: First, activate the column with 10 mL of activation solution. After all the liquid passes through the column, replace the bottom with a clean centrifuge tube and transfer the sample extract to the column. Then, add 2 mL of cyclohexane to wet-clean the centrifuge tube and load until all the liquid passes through the column. Add 5 mL of eluent solution to wash the column, collect the liquid, and then nitrogen-blow and concentrate the liquid at 40 °C to approx. 0.5 mL. Finally, take the sample solution for GC-MS analysis.

## 4. Health Risk Assessment

### 4.1. Toxic Equivalent Content of PAHs

Traditionally, carcinogenic toxicity assessments have been used to evaluate the health risk of an individual’s exposure to a carcinogen. The toxic equivalency assessment of PAHs is based on the toxic equivalency factor (TEF) of benzo [a]pyrene, which is set to 1, and the toxic equivalency of other PAHs is calculated according to the formula of the conversion factor [40,41,42]. The formula for calculating the toxic equivalent quotient (TEQ) concentration of PAHs (TEQBaP) is as follows (1):

(1) $${TEQ}_{BaP}=\frac{C_{i}\times {TEF}_{i}}{1000}$$

where TEQ_BaP_ denotes the toxic equivalence of PAHs converted to benzo[a]pyrene, ng/kg; C_i_ denotes the concentration of PAHs in CHMs, μg/kg; TEF_i_ denotes the TEF value of PAHs in CHMs.

### 4.2. Carcinogenic Risk Assessment

According to the U.S. EPA-recommended Incremental Lifetime Cancer Risk (ILCR) model [43], an ILCR greater than 10^−4^ is considered an unacceptable cancer risk, and results between 10^−6^ and 10^−4^ are classified as potential cancer risks. On the other hand, for ILCR values that are less than 10^−6^, the cancer risk associated with those are considered negligible [44]. The formula for calculating ILCR is shown in Equation (2):

(2) $$ILCR=\frac{{TEQ}_{BaP}\times DR\times CSF\times EF\times ED}{BW\times AT}$$

where DR denotes the daily intake of herbal medicines, which was obtained from the study sample using a questionnaire, CSF denotes the dietary carcinogenicity slop e factor for BaP [45,46]. EF denotes the exposure frequency, taken as 365 d; ED denotes the exposure time; BW is body weight; AT is the average lifetime of the carcinogen [47,48] (see Supplementary Materials Table S1).

### 4.3. Probabilistic Assessment and Sensitivity Analysis

Uncertainty analysis in health risk assessment consists of two main parts: determining the probabilistic outcome and assessing the contribution of each variable to the outcome. Monte Carlo simulation was used to analyze the uncertainty of the results [49]. First, the type of best-fit probability distribution of the exposure factor was simulated by the Anderson–Darling test and chi-squared test, stable exposure distribution results were obtained through 10,000 iterations, and probabilistic risks were assessed by using values of different orders of magnitude (e.g., 10th, 50th, and 90th percentiles) of the exposure distribution results. The extent to which the exposure factors influenced the results was assessed by performing sensitivity analyses. Positive values indicate that the exposure factor is positively associated with health risk; conversely, it is negatively associated [50,51,52].

Determination of the best-fitting distribution for each parameter, Monte Carlo simulation, and sensitivity analysis were performed using Crystal Ball.

## 5. Conclusions

The GC-MS method was employed for the quantitative detection of EPA-PAHs in seven CHMs, and its successful application in analyzing 24 Chinese herbal medicine samples in 2016, with an RSD < 15%, affirmed the method’s accuracy. Extraction and purification steps catered to the diverse components of CHMs were specifically designed, encompassing roots, stems, flowers, fruits, and leaves.

The method’s pretreatment eliminated the need for extensive equipment such as GPS purifiers or bulky laboratory instruments like Soxhlet extraction, reducing solvent usage in the extraction and purification processes and shortening the extraction time. Consequently, this method emerges as a simple, accurate, sensitive, and efficient tool, holding promise for quality control and potential health risk assessment of PAHs in traditional Chinese medicines. Its applicability for online determination of PAHs offers valuable insights for drug safety monitoring and PAH risk management. Experimental results underscore widespread PAH contamination in most herbal medicines, with higher contamination levels observed in flowers and seeds compared to roots and fruits. PAH sources in herbal medicines reveal a combination of fuel oil, biomass, and coal combustion, with wood or coal combustion potentially contributing significantly to PAHs found in root herbs. While honeysuckle consumption may pose a health risk, the consumption of the other six herbs presents negligible health risks.

PAH content in herbs is a contributing factor to health risk sensitivity. Given the potential long-term nature of herbal medicine consumption, especially for patients with chronic diseases, stringent quality control measures are essential. This study not only provides an effective method for quantitative analysis and quality monitoring of PAHs in CHMs, but also offers insights into assessing the pollution levels and health risks associated with herbal medicines, serving as a reference for the safe export and quality development of ethnomedicine.

## Figures and Tables

**Table 1:** Optimized parameters for analysis of PAHs using GC-MS.

Number	Compound	Abbreviation	CAS Number	Molecular Weight	Retention Time (min)	Ion Pair for Quantitative Analysis (*m*/*z*)	Ion Pair for Qualitative Analysis (*m*/*z*)
1	Naphthalene	Naph	91-20-3	128.17	9.273	128 > 102	128 > 77
2	Acenaphthylene	Acy	208-96-8	152.20	12.925	152 > 151	152 > 126
3	Acenaphthene	Ace	83-32-9	154.20	13.326	153 > 152	153 > 127
4	Fluorene	Flu	86-73-7	166.22	14.417	166 > 165	166 > 163
5	Phenanthrene	Phe	85-01-8	178.23	16.345	178 > 176	178 > 152
6	Anthracene	Ant	120-12-7	178.22	16.358	178 > 176	178 > 152
7	Fluoranthene	Flt	206-44-0	202.25	18.549	202 > 200	202 > 150
8	Pyrene	Pyr	129-00-0	202.26	19.028	202 > 200	202 > 150
9	Benz[a]anthracene	BaA	56-55-3	228.30	22.102	228 > 226	228 > 202
10	Chrysene	Chry	218-01-9	228.29	21.971	228 > 226	228 > 202
11	Benzo[b]fluorathene	BbF	205-99-2	252.31	25.551	252 > 250	252 > 224
12	Benzo[k]fluorathene	bkF	207-08-9	252.31	25.640	252 > 250	252 > 224
13	Benzo[a]pyrene	BaP	50-32-8	252.31	26.618	252 > 250	252 > 224
14	Indeno[1,2,3-c,d]pyrene	Ind(cd)P	193-39-5	276.00	30.699	276 > 274	276 > 248
15	Dibenz[a,h]anthracene	DahA	53-70-3	278.35	30.744	278 > 276	278 > 274
16	Benzo[g,h,i]perylene	BghiP	191-24-2	276.33	31.798	276 > 274	276 > 272
17	Naphthalene-d8	Naph-d8	1146-65-2	136.22	9.232	136 > 108	136 > 134
18	Acenaphthene-d10	Ace-d10	15067-26-2	164.17	13.255	164 > 162	136 > 134
19	Phenanthrene-d10	Phe-d10	1517-22-2	188.29	16.213	188 > 160	164 > 160
20	Chrysene-d12	Chry-d12	1719-03-5	240.36	22.016	240 > 236	240 > 212
21	Benzo[a]pyrene-d12	BaP-d12	63466-71-7	266.399	26.788	264 > 260	264 > 232

**Table 2:** Spiked recoveries of 16 PAHs in 7 CHMs (5 PAHs-D as a proxy, *n* = 6).

Analyte	Spiked 20 μg/kg		Spiked 100 μg/kg		Spiked 400 μg/kg	
	Mean Recovery (%)	RSD (%)	Mean Recovery (%)	RSD (%)	Mean Recovery (%)	RSD (%)
Naph	66.97	3.06	79.21	4.38	67.2	0.75
Acy	84.67	4.26	89.37	5.94	84.1	1.54
Ace	63.62	4.91	82.66	3.94	85.4	1.13
Flu	63.37	10.96	76.89	2.28	74.0	2.35
Phe	133.12	2.76	116.05	1.95	108.1	5.21
Ant	116.72	14.54	119.15	9.47	98.9	2.66
Flt	104.45	2.10	104.91	2.74	107.1	3.26
Pyr	113.30	3.27	107.19	3.63	106.4	2.44
BaA	89.27	8.44	121.45	1.04	108.6	4.82
Chry	85.50	13.09	117.37	2.86	99.1	2.50
BbF	119.23	3.89	121.85	6.37	117.0	7.98
bkF	119.15	3.00	117.51	4.49	111.5	2.06
BaP	112.71	14.33	117.96	5.34	113.1	6.39
Ind(cd)P	100.96	4.38	109.01	1.08	114.1	6.18
DahA	106.04	3.31	111.17	5.20	114.2	3.40
BghiP	107.02	0.69	114.56	2.60	116.1	6.00

Note: Relative standard deviation (RSD).

**Table 3:** Analytical performance of the proposed method.

Analyte	Linearity Range (ng/mL)	LOD (μg/kg)	LOQ (μg/kg)	Regression Equation	Coefficient of Determination (R^2^)
Naph	5–500	6.81	13.6	y = 0.453*x* + 0.053	0.9990
Acy	5–500	5.20	10.4	y = 0.626*x* − 0.013	0.9990
Ace	5–500	5.68	11.4	y = 0.759*x* + 0.029	0.9996
Flu	5–500	5.14	10.3	y = 0.517*x* + 0.037	0.9997
Phe	5–500	6.35	12.7	y = 0.527*x* + 0.003	0.9997
Ant	5–500	4.96	9.9	y = 0.571*x* − 0.020	0.9998
Flt	5–500	5.13	10.3	y = 0.474*x* + 0.009	0.9999
Pyr	5–500	5.33	10.7	y = 0.346*x* + 0.025	0.9998
BaA	5–500	5.60	11.2	y = 0.207*x* + 0.032	0.9998
Chry	5–500	5.54	11.1	y = 0.458*x* + 0.009	0.9992
BbF	5–500	6.99	14.0	y = 0.276*x* + 0.005	0.9996
bkF	5–500	6.75	13.5	y = 0.502*x* + 0.017	0.9997
BaP	5–500	8.14	16.3	y = 0.338*x* + 0.016	0.9993
Ind(cd)P	5–500	5.72	11.4	y = 0.440*x* − 0.018	0.9998
DahA	5–500	6.25	12.5	y = 0.245*x* + 0.224	0.9997
BghiP	5–500	7.70	15.4	y = 0.235*x* + 0.019	1.0000

Note: Limit of detection (LOD); limit of quantification (LOQ).

**Table 4:** PAH content in selected CHMs (μg/kg).

Analyte	Glycyrrhizae Radix et Rhizoma	RSD%	Honeysuckle	RSD%	*Coix lacryma*	RSD%	Ginseng Radix et Rhizoma	RSD%	Lotus Seed	RSD%	Seed of *Sterculia lychnophora*	RSD%	*Lycium chinense*	RSD%
Naph	42.313	0.39	36.019	0.25	21.666	0.36	60.153	0.59	37.674	0.40	40.844	0.26	48.876	0.31
Acy	13.600	0.73	13.609	0.32	22.913	0.37	nd	-	18.681	0.55	27.400	0.38	11.378	0.32
Ace	31.851	0.10	25.060	0.56	24.754	0.50	33.842	0.47	27.726	0.52	38.175	0.39	39.044	0.12
Flu	24.576	0.21	25.191	0.51	nd	-	nd	-	nd	-	12.241	0.37	18.880	0.40
Phe	153.753	0.05	160.906	0.21	82.388	0.58	103.442	0.22	80.887	0.30	168.640	0.12	47.045	0.18
Ant	154.849	0.07	nd	-	147.707	0.29	121.351	0.29	117.332	0.23	112.253	0.15	nd	-
Flt	97.790	0.26	52.555	0.17	27.020	0.83	18.462	0.76	21.164	0.64	41.773	0.25	nd	-
Pyr	99.612	0.23	42.752	0.22	nd	-	18.190	0.83	11.859	0.21	52.345	0.40	11.683	0.19
BaA	31.207	0.39	nd	-	nd	-	nd	-	nd	-	nd	-	nd	-
Chry	50.084	0.12	76.563	0.21	nd	-	nd	-	nd	-	nd	-	nd	-
BbF	148.469	0.08	941.504	0.08	nd	-	nd	-	nd	-	462.085	0.04	nd	-
bkF	nd	-	nd	-	nd	-	nd	-	22.594	0.48	nd	-	nd	-
BaP	nd	-	39.928	0.24	nd	-	nd	-	nd	-	nd	-	nd	-
Ind(cd)P	nd	-	nd	-	nd	-	nd	-	nd	-	nd	-	nd	-
DahA	nd	-	nd	-	nd	-	nd	-	nd	-	nd	-	nd	-
BghiP	nd	-	nd	-	nd	-	nd	-	nd	-	nd	-	nd	-
∑16PAHs	848.102	-	1414.087	-	326.448	-	355.440	-	337.917	-	955.758	-	176.906	-

Note: Mean content (μg/kg, *n* = 3, RSD ≤ 15%); nd, not detected; -, excluded from calculation.

**Table 5:** Statistics of probabilistic estimation of lifetime carcinogenic risk values.

CHMs	Distribution	Parameters		10%	50%	90%
		Mean	SD			
Glycyrrhizae Radix et Rhizoma	Lognormal	2.00 × 10^−5^	5.66 × 10^−5^	1.29 × 10^−6^	7.55 × 10^−6^	4.59 × 10^−5^
Honeysuckle	Lognormal	6.04 × 10^−4^	1.48 × 10^−4^	2.96 × 10^−5^	2.00 × 10^−4^	1.37 × 10^−4^
*Coix lacryma*	Lognormal	7.04 × 10^−5^	4.47 × 10^−4^	1.84 × 10^−4^	1.68 × 10^−5^	1.48 × 10^−4^
Ginseng Radix et Rhizoma	Lognormal	5.64 × 10^−5^	1.81 × 10^−4^	1.66 × 10^−4^	1.43 × 10^−5^	1.20 × 10^−4^
Lotus seed	Lognormal	6.56 × 10^−5^	1.98 × 10^−4^	2.45 × 10^−4^	1.93 × 10^−5^	1.45 × 10^−4^
seed of *Sterculia lychnophora*	Lognormal	2.70 × 10^−4^	9.79 × 10^−4^	1.08 × 10^−5^	7.86 × 10^−5^	5.92 × 10^−4^
*Lycium chinense*	Lognormal	5.85 × 10^−7^	1.41 × 10^−4^	−1.03 × 10^−4^	5.53 × 10^−7^	2.25 × 10^−4^

Note: Standard deviation (SD).

## Data Availability

The datasets used and/or analyzed during the current study are available from the corresponding author on reasonable request.

## Supplementary Materials

The following supporting information can be downloaded at: https://www.mdpi.com/article/10.3390/molecules29050972/s1, Table S1. Calculation parameters for lifetime cancer risk assessment. Table S2. Commonly used PAHs characterization ratios and their sources. Table S3. Health Risk Assessment Results of 7 Chinese Herbal Medicines (ILCR).
