# Supplementary material for: Concentrations, Sources and Health Risk Assessment of Polycyclic Aromatic Hydrocarbons in Chinese Herbal Medicines
Source: Molecules. 2024 Feb 22;29(5):972. doi: 10.3390/molecules29050972 (PMC10934209; doi:10.3390/molecules29050972)
Supplement: Supplementary file 1 [file molecules-29-00972-s001.zip › molecules-2848239-supplementary.pdf]

Supplementary Material

Concentrations, Sources and Health Risk Assessment of Polycyclic Aromatic Hydrocarbons in Chinese Herbal Medicines

Deyan Cao[1,2], Zhu Zhu[1,2], Siyuan Zhao[1,2], Xi Zhang[3], Jianzai Lin[1,2], Junji Wang[1,2], Qinghong Zeng[1,2], Meilin Zhu[1,3*]

1 School of Public Health, Ningxia Medical University, Yinchuan 750004, China
2 Key Laboratory of Environmental Factors and Chronic Disease Control, Ningxia Medical University, Yinchuan 750004, China
3 College of Basic Medical Sciences, Ningxia medical University, Yinchuan 750004, China

***Corresponding Author:**
Meilin Zhu

Ningxia Medical University
Email: 20140127@nxmu.edu.cn

Table S1 Calculation parameters for lifetime cancer risk assessment

Parameter	Meaning	Unit	Value			
			Children	Teenagers	Adults	Seniors
DR	Daily intake of herbal medicines	mg/kg	Questionnaire survey (survey of 400 Chinese residents of different age groups)			
CSF	Dietary carcinogenicity slop e factor for BaP	mg/kg· d	7.3			
EF	Exposure frequency	d	365			
ED	Exposure time(year)		7	7	43	10
BW	Body weight	mg/kg·d	16.40	36.40	59.70	60.60
AT	Average lifetime of the carcinogen	d	25550			

Table S2 Commonly used PAHs characterization ratios and their sources

Specific value	Estimation 1	Estimation 2	Estimation 3
Ant/(Ant + Phe)	≤0.1,crude oil contamination	＞0.1,Source of combustion	

Flt/(Flt + Pyr)	≤0.4,crude oil contamination	＞0.5,Biomass combustion sources	0.4-0.5,Gasoline combustion sources
BaA/(BaA + Chr)	≤0.2,crude oil contamination	＞0.35,Biomass or coal combustion source	0.20-0.35,Sources of oil combustion

Table S3 Health Risk Assessment Results of 7 Chinese Herbal Medicines (ILCR)

Age groups	Glycyrrhizae Radix Et Rhizoma	Honeysuckle	Coix lacryma	Ginseng Radix Et Rhizoma	Lotus seed	Seed of Sterculia lychnophora	Lycium chinense
Children	7.11×10^{-5}	3.69×10^{-4}	1.28×10^{-4}	4.87×10^{-5}	1.20×10^{-4}	3.21×10^{-5}	5.00×10^{-7}
Teenagers	3.20×10^{-5}	1.66×10^{-4}	5.75×10^{-5}	2.20×10^{-5}	5.43×10^{-5}	1.45×10^{-5}	2.25×10^{-7}
Adults	1.20×10^{-4}	6.23×10^{-4}	2.15×10^{-4}	8.22×10^{-5}	2.03×10^{-4}	5.42×10^{-5}	8.43×10^{-7}
Seniors	2.75×10^{-5}	1.43×10^{-4}	4.93×10^{-5}	1.88×10^{-5}	4.66×10^{-5}	1.24×10^{-5}	1.93×10^{-7}
